# Peripheral Thromboembolism Formation in a Case of Takotsubo Cardiomyopathy

**DOI:** 10.7759/cureus.24087

**Published:** 2022-04-12

**Authors:** Reema S Patel, Paige Webeler, Uma Mahesh Gudur

**Affiliations:** 1 Internal Medicine, Nova Southeastern University Dr. Kiran C. Patel College of Osteopathic Medicine, Fort Lauderdale, USA; 2 Internal Medicine, AdventHealth Ocala, Ocala, USA

**Keywords:** angiography, catecholamine excess, apical ballooning, thromboembolism, stress-induced cardiomyopathy, broken heart syndrome, takotsubo cardiomyopathy

## Abstract

Takotsubo cardiomyopathy (TC) is a reversible cardiac disorder of the elderly commonly seen in postmenopausal women. TC can present similar to acute coronary syndrome with chest pain and dyspnea; however, there is no coronary artery occlusion. Patients with TC classically show ventricular apical ballooning and hypokinesis on echocardiogram. Few cases of thromboembolism of the lower extremity due to Takotsubo have been reported in the literature. Here, we report a unique occurrence of a thromboembolism formation in the right common iliac and external iliac arteries in the context of TC. Through the mechanisms of TC that involve ventricular wall abnormalities, hypercoagulability, and catecholamine excess, thromboembolism in the iliac system was likely from the akinetic ventricle. In this case, computed tomography angiography was an essential diagnostic tool that revealed the peripheral occlusion; therefore, it should be considered in patients with a history of TC who complain of lower extremity pain and neurological symptoms.

## Introduction

Takotsubo cardiomyopathy (TC) is a reversible cardiac disorder commonly occurring in postmenopausal women discovered in the early 1990s in Japan [1]. The syndrome presents similar to acute coronary syndrome (ACS) with chest pain and dyspnea. Rather than coronary artery occlusion, patients are found to have ventricular apical ballooning and hypokinesis on echocardiogram [2]. TC is thought to be caused by catecholamine excess from significant physical or emotional stress [3]. Although this disorder usually resolves independently, serious complications can occur from ventricular dysfunction such as shock, heart failure, and thromboembolism [3]. Few cases of thromboembolism in a large peripheral artery due to TC have been reported in the literature. We present this unique case to raise awareness regarding the possibility of peripheral thromboembolism formation as a complication of TC to improve patient outcomes.

## Case presentation

A 78-year-old Caucasian female with a medical history of chronic obstructive pulmonary disease (COPD), hypertension, hypothyroidism, gastroesophageal reflux disease (GERD), chronic pulmonary *Mycobacterium avium-intracellulare* (MAI) infection, and recent myocardial infarction (MI), as well as recently diagnosed TC and congestive heart failure (CHF), presented to the emergency department complaining of chest pain and dyspnea. She did not have any known allergies. She was a former smoker. Medications taken before admission included 2.5 mg amlodipine oral tablet once daily for hypertension, 500 mg calcium carbonate oral tablet twice daily, 200 µg levothyroxine daily for hypothyroidism, omeprazole oral capsule daily for GERD, 50 mg metoprolol oral tablet twice daily for CHF, and azithromycin and rifampin for MAI infection.

In the emergency room, the following vital signs were recorded: temperature of 97.8°F, pulse of 70 beats/minute, blood pressure of 113/49 mmHg, respiratory rate of 18 breaths/minute, and oxygen saturation of 93% on room air. She was alert, oriented, and in no acute distress but cachectic because she had been losing weight recently. Her eye examination showed pupils that were equally round and reactive to light with intact extraocular movements. Conjunctiva appeared to be normal. No lymphadenopathy was noted in the neck, axilla, or inguinal areas. The head was normocephalic. Ear, nose, and throat examinations were normal with clear tympanic membranes, normal hearing, moist oral mucosa, no scleral icterus, and no sinus tenderness. The neck was supple, non-tender with no carotid bruits, and no lymphadenopathy; however, a 3 cm jugular venous distension was noted. Lung examination revealed poor vesicular breath sounds, prolonged expiration, and occasional rales in the bases without any wheezing. The heart was regular in rate and rhythm with an audible S1, S2 with intermittent S3, P2 louder than A2 with no murmurs, no rubs, no gallops, and trace peripheral edema. Abdominal examination showed a soft, non-tender, non-distended abdomen with normal bowel sounds and no masses or organomegaly. Extremity examination revealed a normal range of motion and strength with trace pedal edema. The skin was warm, dry, and pink without rashes. The patient was oriented to person, place, and time with intact cranial nerves II-XII and no focal signs. The patient was cooperative with an appropriate mood and affect.

Initial laboratory studies revealed critical troponin T of 83 ng/L followed by 79 ng/L (normal ≤14). Troponin T levels on the next day after admission remained elevated at 78 ng/L. Initial pro-B-type natriuretic peptide (pro-BNP) was determined to be high at 14,107 pg/mL (normal <450 pg/mL for patients aged 75-79 years [4]).

**Figure 1:**
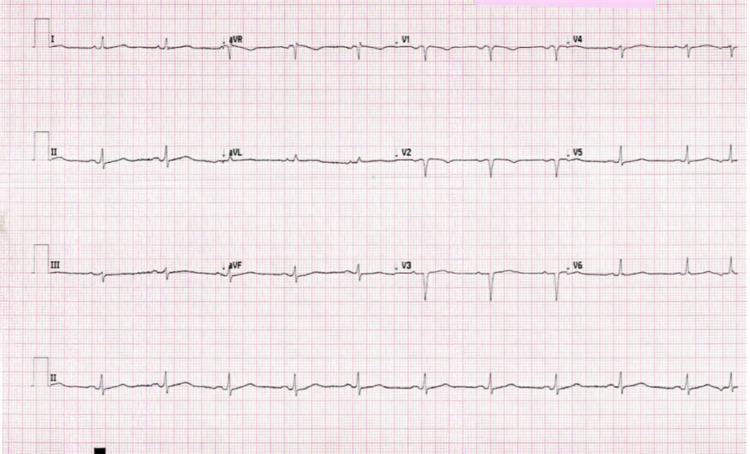
Echocardiogram showing sinus rhythm, poor R wave progression with Q waves, a premature atrial contraction, biphasic P waves, and low voltage.

The initial electrocardiogram showed sinus rhythm, poor R wave progression with Q waves in all anterior leads, a premature atrial contraction, and low voltage. It also demonstrated evidence of left atrial enlargement with a biphasic P wave in lead II and a P wave with a negative deflection in lead V1. No ST-elevation or depression was noted (Figure 1). Cardiac catheterization showed no evidence of occlusive coronary artery disease. Echocardiography (ECHO) showed an ejection fraction of 40%, mild mitral valve regurgitation, mild tricuspid valve regurgitation, and mild enlargement of the right ventricle with moderate hypokinesis. Computed tomography angiography (CTA) of the chest with contrast showed bilateral pleural effusions and decompensated CHF with no signs of pulmonary embolism.

**Figure 2:**
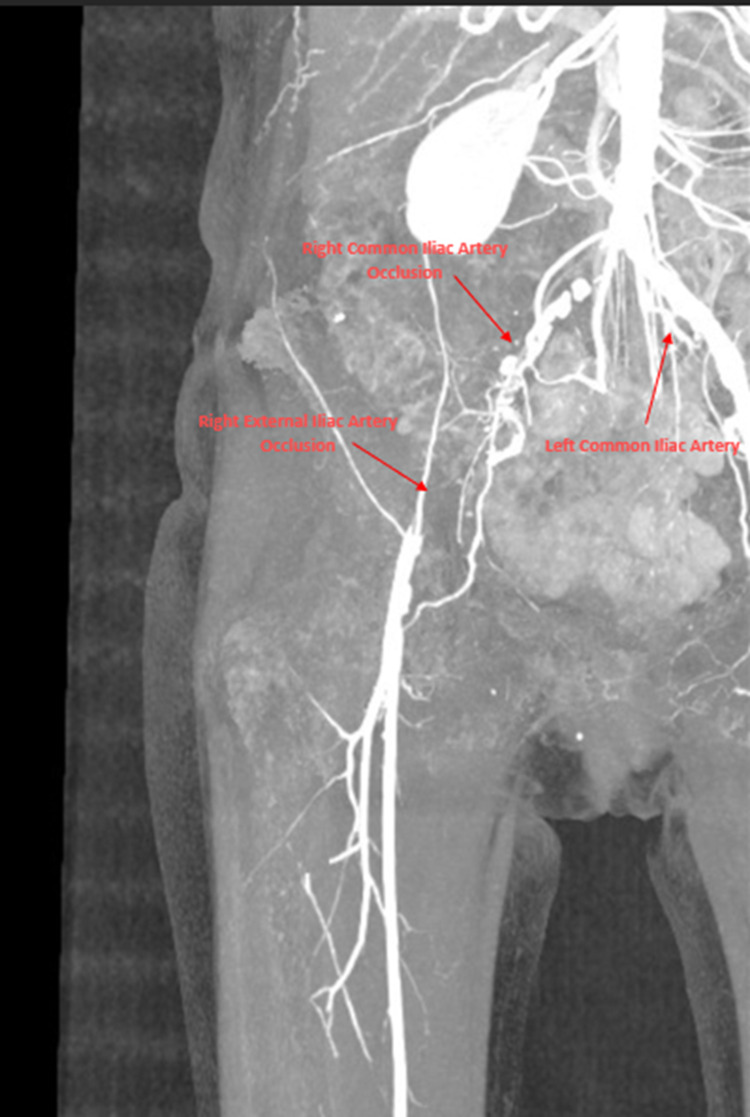
CTA of the abdominal aorta with runoff showing occlusion of the right common and right external iliac arteries. CTA: computed tomography angiography

**Figure 3:**
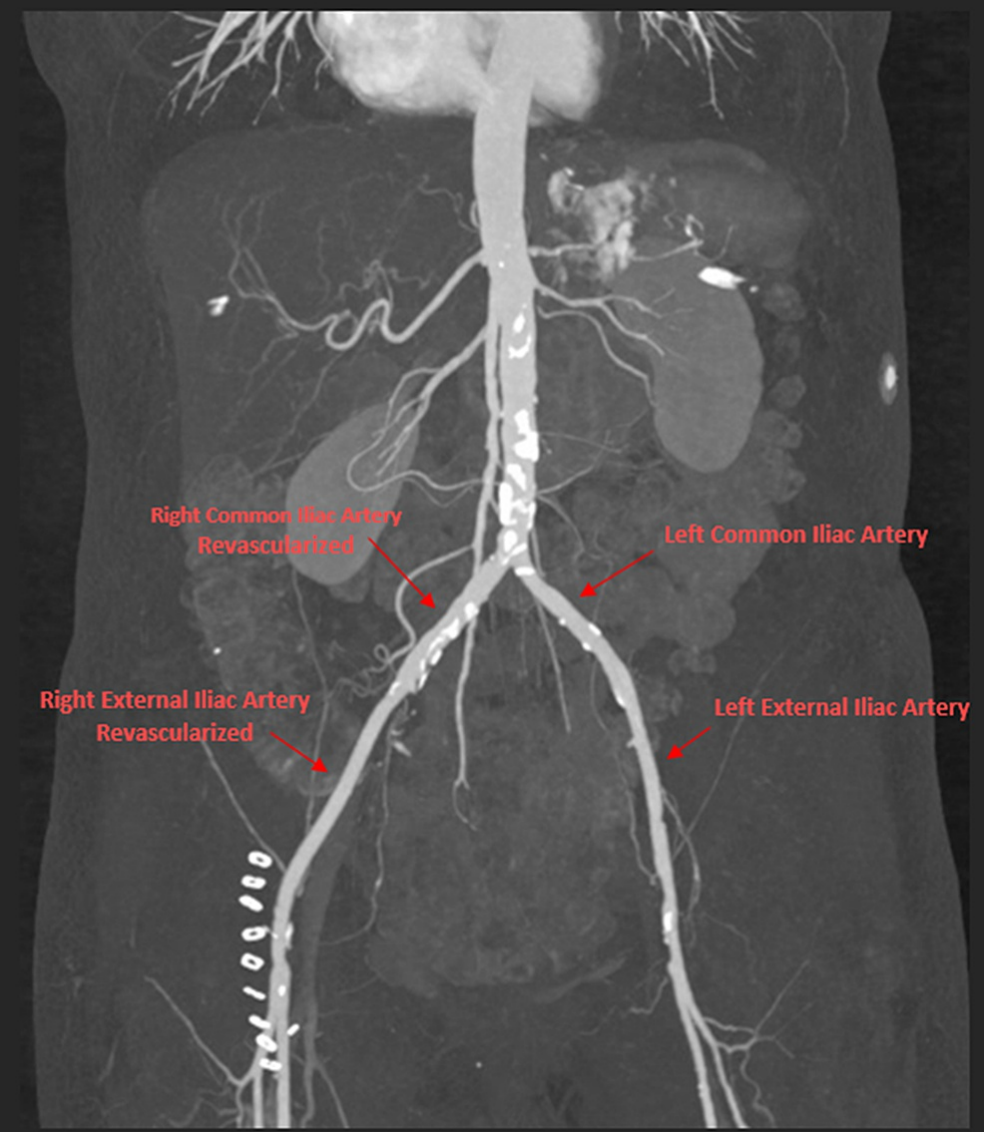
CTA of the abdominal aorta with runoff after thrombectomy showing revascularization of the right common and right external iliac arteries. CTA: computed tomography angiography

During the first day after admission, the patient complained of right lower extremity weakness. Magnetic resonance imaging (MRI) of the lumbar spine showed degenerative changes with mild stenosis at L3-L4 and moderate changes at L4-L5. Physical examination revealed right lower extremity numbness with decreased right foot dorsiflexion and eversion. Vascular examination indicated the absence of popliteal and pedal pulse in the right lower extremity. Intravenous (IV) heparin (80 U/kg) was administered. CTA of the abdominal aorta with runoffs was used to visualize the patency of the abdominal aorta and distal arterial branches of the lower extremity. It revealed right common and external iliac artery occlusion with good distal reconstituted flow (Figure 2). Right iliac and femoral artery thrombectomy revealed stenosis of the common iliac artery distal to the proximal external iliac artery. Thrombi in the iliac arterial system including the superficial femoral artery and profundus femoral artery were removed. The right lower extremity improved in sensation, perfusion, warmth, and 5/5 strength status post-thrombectomy. CTA of the abdominal aorta with runoff after thrombectomy revealed restoration of patency in the right common and external iliac arteries (Figure 3). The patient was put on anticoagulant therapy with Lovenox until the ejection fraction improved. Per cardiology, there was a high suspicion of thrombus embolization from the left ventricular apex given the recent history of TC. Transesophageal echocardiogram (TEE) after thrombectomy was negative for thrombus or valvular vegetation but revealed an akinetic left ventricular apex with moderately decreased ejection fraction.

## Discussion

TC is a reversible cardiac syndrome primarily found in postmenopausal women that was discovered in the early 1990s in Japan [1]. TC is thought to be triggered by a surge in catecholamines from stressful physical or emotional events and can be categorized into primary or secondary based on clinical history. In primary TC, patients present with cardiac symptoms that are likely the result of emotional stress. Secondary TC results from a pre-existing medical condition, illness, or surgery that triggers a rise in catecholamines [5].

The circulating catecholamines are thought to lead to both myocardial and vascular complications such as myocardial stunning, coronary vasospasm, left ventricular outflow tract obstruction, and increased afterload [5]. Iatrogenic sympathetic agonists such as epinephrine or dobutamine have been found to induce apical ballooning and ventricular dysfunction seen in TC [6]. It is believed that catecholamines damage the structure of the myocytes leading to alteration in overall shape and contractile function [7]. TC can also be idiopathic with no known physical or emotional trigger. This could be the case with our patient as she reported no recent emotional or physical events. However, she had a chronic MAI complex infection with worsening weight loss over the past few months. This infection could have hit a threshold where the stress on the body triggered a release of catecholamines, initiating the TC [1].

Symptoms of TC are similar to ACS. However, there is no coronary artery occlusion. TC is the diagnosis in 1-3% of individuals presenting for possible ACS [1]. Although a formal diagnosis criterion for TC has not yet been established, the modified criteria from Mayo Clinic are often used to make a diagnosis. The four main criteria include (1) transient left ventricular wall motion abnormalities such as akinesis, dyskinesis, and hypokinesis; (2) absence of obstructive coronary artery disease; (3) new electrocardiogram abnormalities such as ST-elevation, T wave inversion, or elevated troponins; and (4) absence of pheochromocytoma or myocarditis [2].

There were wall motion abnormalities of anatomical regions supplied by more than one coronary artery in the patient presented. There was moderate hypokinesis of the right ventricle seen on the ECHO and akinesis of the left ventricle noted from TEE. In line with the Mayo Clinic criteria for TC, the cardiac catheterization report in our patient demonstrated no occlusive coronary artery disease. In addition, troponin levels in the emergency department (83 ng/L) and upon admission (79 ng/L) were significantly elevated (normal ≤14). These levels are thought to be elevated due to myocardial damage and coronary artery vasospasm from catecholamine excess that occurs in TC [7]. Lastly, there were no signs of pheochromocytoma or myocarditis suspected in this patient. Cumulatively, these attributes indicated that the patient was experiencing an exacerbation of her recent diagnosis of TC. It is also important to note that BNP elevation is almost universal in TC, and the magnitude of elevation is more significant than in acute MI [8]. Elevated BNP is indicative of fluid overload, as seen in ventricular dysfunction and heart failure. In our patient, the BNP levels were 14,107 ng/mL, more than 30 times the upper limit of normal for the patient’s age group (age 75-79 years) for which the BNP should be <450 pg/mL.

Although various diagnostic studies can be used to diagnose TC, an ECHO remains the most informative for evaluating ventricular and valvular function. The left ventricular apical ballooning is pathognomonic on ECHO and is what gave Takotsubo its name. The ballooning was thought to resemble the shape of the Japanese octopus trap called Takotsubo [2]. Other names include broken heart syndrome and stress-induced cardiomyopathy, derived from the most likely cause of the disorder. Although the exact cause of TC is not known, catecholamine-induced cardiotoxicity from physical or emotional stress is thought to be the most common trigger [9]. This stress can include positive and negative emotions; other possible causes include coronary vasospasm or vascular dysfunction [3]. A recent study found an increased incidence of TC during the pandemic in people without coronavirus disease 2019. The rise is thought to be from the increased psychological stress of the pandemic [10].

Classically, the left ventricle is solely involved in TC but wall motion abnormalities of the right ventricle can occur as well. Patients with biventricular TC experience worse left ventricular ejection fractions (LVEFs) and an increased incidence of pleural effusions [11]. There is evidence of purely right ventricular TC in some instances [5]. Our patient presented with evidence of both left and right ventricular dysfunction on an ECHO during her hospital stay. She had a low ejection fraction and bilateral pleural effusions consistent with the involvement of the right ventricle.

Clinical management for TC generally entails supportive therapy as left ventricular function normalizes over time. Patients are usually monitored on telemetry for arrhythmias. ECHO can be helpful to assess left ventricular function after an episode of TC. The ECHO in our patient revealed a 40% ejection fraction with a mildly enlarged right ventricle and moderate hypokinesis of the right ventricle. There is a lack of evidence from randomized trials for a proven management algorithm for TC based on a consensus. Thus, there is a need for prospective trials to help guide clinical diagnosis and management for these patients. Due to high-risk complications that can arise from TC, it is encouraged to admit patients to a cardiac care unit with electrocardiogram monitoring for the first 24 hours [5]. In cases of TC with an LVEF of >45%, medications should be reviewed and patients may be started on ACS treatment, antiplatelet agents, or statins if necessary. If LVEF is 35-45%, angiotensin-converting enzyme inhibitors can be used to alleviate heart failure symptoms, and beta-blockers should be considered to reduce sympathetic drive. In this case, the patient was on metoprolol before arrival at the hospital and throughout her stay.

In severe cases of TC, patients should be admitted to a cardiac intensive care unit with electrocardiogram monitoring and resuscitation equipment for the first 72 hours. If cardiopulmonary resuscitation is required, equipment such as oxygen masks, oxygen, automated external defibrillator devices, and epinephrine should be available. Complications such as cardiogenic shock, arrhythmia, or left ventricular outflow obstruction should be treated as they usually would be. However, the use of sympathomimetic drugs should be minimized [12]. Treatment with short-acting beta-blockers or selective alpha agonists should be considered unless clinical features of pheochromocytoma are noted. In patients with signs of cardiogenic shock, mechanical support with temporary left ventricular assist devices and extracorporeal membrane oxygenation or levosimendan infusion can be considered [5]. Levosimendan is a non-catecholamine-positive inotrope that increases the calcium sensitivity of cardiomyocytes ultimately improving myocardial contractility in the setting of heart failure [13]. An ECHO is recommended before discharge from the hospital to determine the latest ejection fraction. This should be reassessed in one to two months for return to baseline. The systolic function usually recovers in one to three months. Anticoagulation therapy is used in patients with a known thrombus seen on ECHO or in patients with a low ejection fraction [12]. TC patients should be followed for six months after discharge.

TC usually resolves with supportive treatment over days to weeks. However, serious complications can arise. These include cardiogenic shock, heart failure, and thromboembolism. The apical ballooning and hypokinesis of the left ventricle can lead to a decreased ejection fraction which can cause cardiogenic shock and heart failure. Systolic heart failure is present in approximately 12-45% of patients and is especially common in those admitted with highly elevated troponins, advanced age, low ejection fraction, and physical stressors. Stroke is a risk from TC due to the potential for thrombus formation in the akinetic left ventricle. Thrombus forms in about 2-8% of cases [14]. Ventricular thrombus can form due to Virchow’s triad (stasis, endothelial damage, hypercoagulable state). The akinetic ventricle contributes to blood stasis and catecholamines are thought to contribute to endothelial damage and a hypercoagulable state [15]. Thrombus formation most commonly occurs two to five days after symptom onset [14]. This thrombus can embolize and result in a stroke, pulmonary embolism, or can lodge in the peripheral arteries. Our patient developed right common and external iliac artery occlusion from thromboembolism which presented as right lower extremity weakness, numbness, and decreased right foot dorsiflexion within two days of onset of symptoms.

Due to the rarity of TC, data regarding the frequency of recurrence is limited. Recurrence rates in five years ranged from 5% to 22% with a subsequent episode occurring between three months to 10 years after the initial event [5]. Although most patients recover from TC without significant complications, the physiologic abnormalities in cardiac function can persist and cause dyspnea, palpitations, and angina in states of increased sympathetic activity. The data on long-term survival rates are currently limited but there does seem to be an increasing chance of mortality in the first four years after the initial diagnosis of TC [5].

## Conclusions

We report a unique case of thromboembolism formation in the right common iliac and external iliac arteries in the context of TC. Patients with TC are predisposed to thrombus formation in the heart due to ventricular wall abnormalities, hypercoagulability, and catecholamine excess, making it likely that the thromboembolism originated in the heart. It is crucial for healthcare professionals to be aware of the potential of thrombus formation in large arteries in patients with TC. In this case, the CTA was an essential diagnostic tool that revealed the peripheral occlusion; therefore, it should be considered in patients with a history of TC who complain of lower extremity pain and neurological symptoms.
